# Lupus enteritis: from clinical findings to therapeutic management

**DOI:** 10.1186/1750-1172-8-67

**Published:** 2013-05-03

**Authors:** Peter Janssens, Laurent Arnaud, Lionel Galicier, Alexis Mathian, Miguel Hie, Damien Sene, Julien Haroche, Catherine Veyssier-Belot, Isabelle Huynh-Charlier, Philippe A Grenier, Jean-Charles Piette, Zahir Amoura

**Affiliations:** 1 Department of internal medicine, French reference centre for Systemic Lupus Erythematosus, AP-HP, Hôpital Pitié-Salpêtrière, F-75013, Paris, France; 2 Department of internal medicine, Universitair Ziekenhuis Brussel, Vrije Universiteit Brussel, Brussels, Belgium; 3 Institut National de la Santé et de la Recherche Médicale (INSERM), UMR-S 945, Paris, France; 4 Department of clinical immunology, Hôpital Saint Louis, Paris, France; 5 Université Paris Diderot, Sorbonne Paris Cité, Paris, France; 6 Université Pierre et Marie Curie, UPMC Univ Paris 06, F-75005, Paris, France; 7 Department of internal medicine, Hôpital Lariboisière, Paris, France; 8 Department of internal medicine and nephrology, Centre Hospitalier Intercommunal de Poissy Saint-Germain-en-Laye, Saint-Germain-en-Laye, France; 9 Department of radiology, Hôpital Pitié-Salpêtrière, Paris, France

**Keywords:** Systemic lupus erythematosus, Abdominal pain, Lupus enteritis, Small bowel disease, Vasculitis

## Abstract

Lupus enteritis is a rare and poorly understood cause of abdominal pain in patients with systemic lupus erythematosus (SLE). In this study, we report a series of 7 new patients with this rare condition who were referred to French tertiary care centers and perform a systematic literature review of SLE cases fulfilling the revised ACR criteria, with evidence for small bowel involvement, excluding those with infectious enteritis. We describe the characteristics of 143 previously published and 7 new cases. Clinical symptoms mostly included abdominal pain (97%), vomiting (42%), diarrhea (32%) and fever (20%). Laboratory features mostly reflected lupus activity: low complement levels (88%), anemia (52%), leukocytopenia or lymphocytopenia (40%) and thrombocytopenia (21%). Median CRP level was 2.0 mg/dL (range 0–8.2 mg/dL). Proteinuria was present in 47% of cases. Imaging studies revealed bowel wall edema (95%), ascites (78%), the characteristic target sign (71%), mesenteric abnormalities (71%) and bowel dilatation (24%). Only 9 patients (6%) had histologically confirmed vasculitis. All patients received corticosteroids as a first-line therapy, with additional immunosuppressants administered either from the initial episode or only in case of relapse (recurrence rate: 25%). Seven percent developed intestinal necrosis or perforation, yielding a mortality rate of 2.7%. Altogether, lupus enteritis is a poorly known cause of abdominal pain in SLE patients, with distinct clinical and therapeutic features. The disease may evolve to intestinal necrosis and perforation if untreated. Adding with this an excellent steroid responsiveness, timely diagnosis becomes primordial for the adequate management of this rare entity.

## Introduction

Abdominal pain is a frequent symptom in patients diagnosed with systemic lupus erythematosus (SLE) [1]. Apart from the classic causes of acute abdomen, the physician should be aware of infectious complications linked to immunosuppressive treatments as well as more disease-specific conditions such as pancreatitis [2,3], intestinal pseudo-obstruction [4] and lupus enteritis (Table 1) [5]. The frequency of this complication is currently unknown, as lupus enteritis has been reported to be either the most common [6] or contrarily a rare [7] cause of abdominal pain in SLE patients. Furthermore, nomenclature is confusing, with lupus enteritis, mesenteric arteritis, intestinal vasculitis, enteric vasculitis, mesenteric vasculitis, lupus peritonitis and abdominal serositis among others used to name seemingly the same condition [8]. In the BILAG 2004, lupus enteritis is defined as either vasculitis or inflammation of the small-bowel, with supportive image and/or biopsy findings, which underlines the broad spectrum of the disease. Therefore, lupus enteritis should be considered a poorly defined cause of abdominal pain in SLE. Here, we report 7 new cases of lupus enteritis and perform a systematic review of the literature to describe in depth the pathogenic, clinical, laboratory and radiological aspects of this rare SLE feature, as well as the response to treatment and long-term follow-up.

**Table 1:** Leading causes of acute abdominal pain in SLE patients

**Non-SLE related**	**SLE related**
Appendicitis	Lupus enteritis
Lithiasic cholecystitis	Pancreatitis
Peptic ulcer	Pseudo-obstruction
Acute pancreatitis	Acalculous cholecystitis
Retroperitoneal hematoma	Mesenteric thrombosis
Ovarian pathology	Hepatic thrombosis
Diverticulitis	Medication (NSAIDs, MMF, steroids, HCQ…)
Adhesions, intestinal occlusion	Colon perforation (vasculitis)
Infectious Enteritis	
Pyelonephritis	
CMV colitis	

SLE: Systemic Lupus Erythematosus; NSAIDs: Nonsteroidal anti-inflammatory drugs; MMF: mycophenolate mofetil; HCQ: hydroxychloroquine.

### Study design and patient selection

The current multicenter retrospective study is based on 7 consecutive patients with lupus enteritis, referred to two tertiary care centers (department of internal medicine, Hôpital Pitié-Salpêtrière and department of clinical immunology, Hôpital Saint Louis, Paris, France) between January 1990 and December 2011. These patients were identified from computerized databases, which records the main diagnoses for each patient, and from an electronic review of all medical records. The databases were searched for the International Classification of Diseases 10th Revision (ICD-10) code for SLE (code M32) as well as for “Lupus enteritis”. Additional cases were contributed after hand-search of patient files. The medical records of all patients identified were reviewed by 2 physicians (PJ and LA) to ensure that patients fulfilled the revised ACR criteria for SLE [9,10] and had clinical and radiological evidence for small bowel involvement (Small bowel wall edema, abnormal bowel-wall enhancement [double halo or target sign], dilatation of bowel lumen and mesenteric abnormalities such as engorgement of mesenteric vessels, increased number of visible vessels [comb’s sign], and increased attenuation of mesenteric fat). Patients with infectious causes of enteritis were excluded.

### Data collection

We collected data using a form specifically designed for this study, recording information about demographics, comorbidities, clinical history of lupus enteritis, imaging, laboratory data, histology, treatment and outcome.

### Literature review

We also performed a systematic literature review by searching PubMed for articles published between 1964 and July 2012, combining the Mesh terms “systemic lupus erythematosus”, “enteritis”, “vasculitis”, “serositis”, “digestive system”, “intestines” and “mesentery”. We further searched the reference lists of identified articles for additional papers. All papers identified were reviewed by 2 physicians (PJ and LA) to ensure that all patients fulfilled the revised ACR criteria for SLE and had clinical and radiological evidence for small bowel involvement (as described above). Case reports on infectious causes of enteritis and papers with insufficient clinical information were excluded.

### Statistical analysis

Quantitative variables were expressed as mean and standard deviation or as median and range while qualitative variables were expressed as numbers and percentages. Statistical analysis was performed using GraphPad Prism, version 5.0 (GraphPad, San Diego, CA).

### Case reports

#### Case #1

An 18-year old female was diagnosed in 2003 with lupus enteritis on basis of acute abdominal complaints and a typical target image (small bowel wall edema with abnormal bowel-wall enhancement) on abdominal CT-scan. At that time she received prednisolone 5 mg and HCQ 400 mg on a daily basis as she had been diagnosed with lupus 2 years earlier based on cutaneous involvement, cytopenia, and presence of antinuclear antibodies with positive search for anti-dsDNA antibodies. She was administered IV methylprednisolone with rapid improvement. She was recently reevaluated for recurrent abdominal pain and nausea. Physical examination on admission was unremarkable. Biology showed a lupus anticoagulant (without history of thrombosis or abortion), positive ANA and ds-DNA without other signs of active SLE (SLEDAI 1). A CT scan was normal. Treatment was unchanged. No recurrence could be objectified until present.

#### Case #2

A 31 year-old female had been diagnosed with lupus in 2000 when she presented with arthritis, malar rash, pericarditis, positive ANA and ds-DNA antibodies. In 2006, she developed acute abdominal pain with nausea and vomiting. At that time, her daily treatment consisted of prednisone 10 mg per day and HCQ 400 mg per day. She presented no clinical signs of lupus activity (SLEDAI 3). An abdominal CT-scan revealed small bowel edema with target sign. She received IV methylprednisolone (1 g per day for 2 days) and tapered oral prednisone, with prompt clinical and radiological recovery (the follow-up CT-scan at 10 days was normal). However, she presented a similar episode 8 months later while on prednisone 5 mg per day and HCQ. She was again administered IV methylprednisolone (1g per day for 3 days), and a long-term treatment with MMF 2 g per day was initiated. MMF was halted in 2008 because of a pregnancy wish, with no recurrence of lupus enteritis until present.

#### Case #3

**Figure 1:**
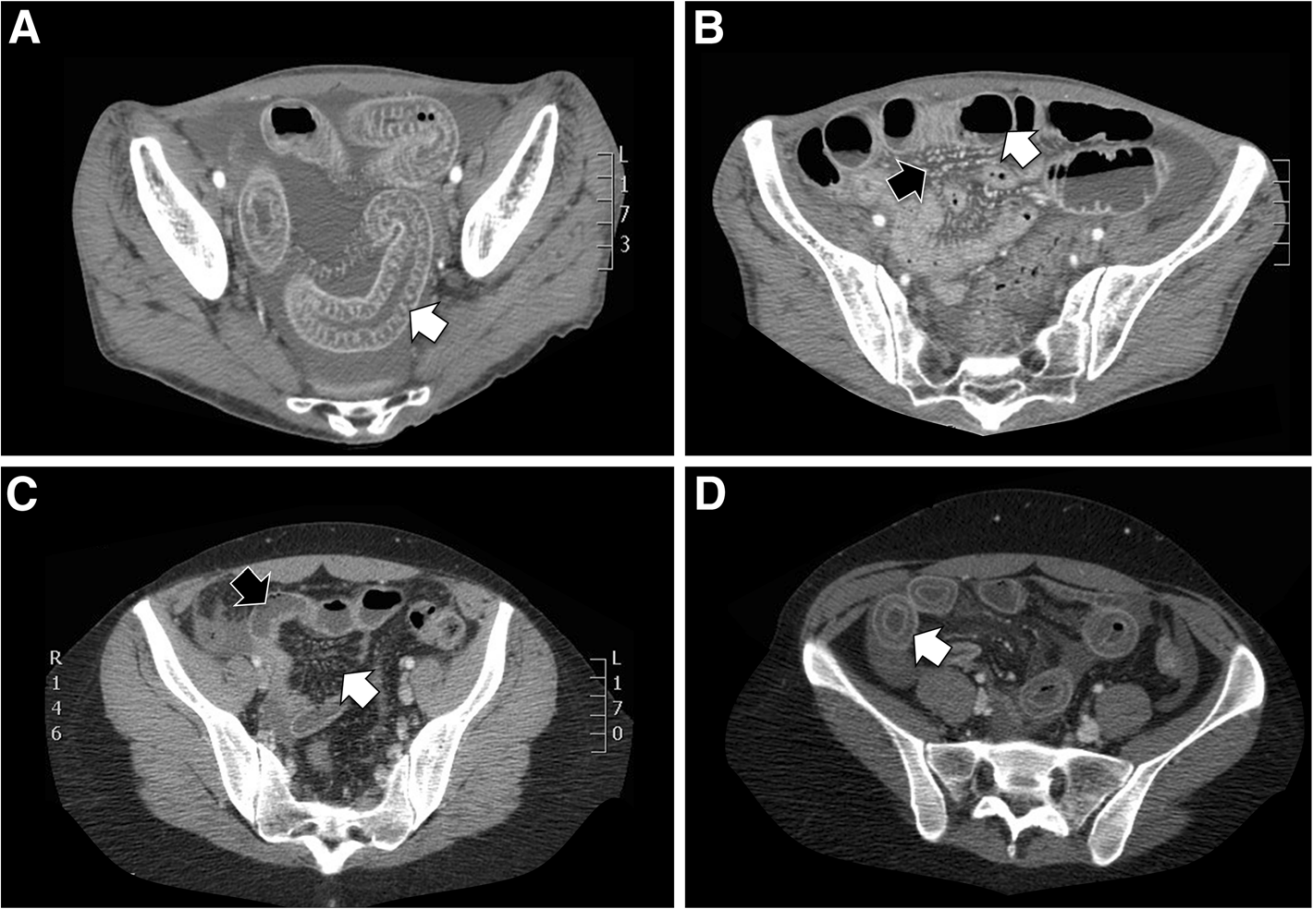
**Imaging findings of lupus enteritis.** Panel **A**: Edematous small-bowel wall (ileum) with the characteristic target sign (abnormal bowel-wall thickening and enhancement) and ascites. Panel **B**: Small bowel distention with engorgement of mesenteric vessels and ascites. Panel **C**. Edematous ileum wall with engorgement of mesenteric vessels and increased number of visible vessels (comb’s sign). Panel **D**: Bowel-wall thickening and enhancement (target sign). All the above described abnormalities are non-specific and can also be seen in patients with pancreatitis, mechanical bowel obstruction, peritonitis, or inflammatory bowel disease, all of which may mimic intestinal ischemia.

A 47 year-old female, diagnosed with SLE at the age of 43, was admitted in 2007 with anorexia, weight loss and a 1-month history of asthenia, intermittent fever, nausea, vomiting and arthralgia. In this patient, SLE was diagnosed based on a history of arthritis, oral ulcers, lupus nephritis, and presence of antinuclear antibodies with positive search for anti-dsDNA antibodies. At admission, her abdomen was mildly tender. Relevant biology results included lymphocytopenia (1472/mm^3^), positive antinuclear antibody (ANA) at 1:1600, highly elevated anti ds-DNA (> 300 U/mL, normal range 0–27 U/mL), low serum albumin (28 g/L), normal C3 complement fraction (0.78 g/L, normal range: 0.72-1.39 g/L), low C4 (0.09 g/L, normal range; 0.15-0.32 g/L) and CH50 (63%, normal range: 70-130%), increased proteinuria (2.25 g/L) with normal creatinine value, and suppressed TSH; C-reactive protein (CRP) were normal, antiphospholipid (aPL) antibodies were negative. She was diagnosed with SLE flare with class III + V lupus nephritis and associated Graves hyperthyroidism. The Systemic lupus erythematosus disease activity (SLEDAI) score was 19. She was started on IV methylprednisolone (1g per day for 3 days) and a concomitant first dose of IV cyclophosphamide (CYC) was administered, followed by oral prednisone (1 mg/kg/day). Four days later, she developed acute abdominal pain and vomiting. Abdominal CT-scan revealed thickened wall with target sign of the terminal ileum, ascites and peritoneal enhancement after intravenous (IV) contrast (Figure 1). Concomitant lupus enteritis was diagnosed and IV methylprednisolone was resumed (40 mg per day). Abdominal pain disappeared over the next three days and oral prednisone could be reinstated six days later. She further received 11 monthly cycles of CYC (IV 750 mg/m^2^) for lupus nephritis. Finally, she regained weight and on maintenance therapy with hydroxychloroquine (HCQ) 400 mg per day, mycophenolate mofetil (MMF) 2 g per day and oral prednisone slowly tapered to 5 mg per day no recurrence of abdominal pain was observed until present (December 2011).

#### Case #4

In 2008, a 27-year old female was transferred because of severe generalized edema that developed 1 month after delivery at 35 weeks of a healthy girl. In this patient, diagnosis of SLE was based on arthritis, cytopenia, and presence of antinuclear antibodies with positive search for anti-dsDNA antibodies. She had been also diagnosed with class V lupus nephritis in 2007 and treated with MMF and cyclosporine that were interrupted 3 months later because of pregnancy. On admission, she was on prednisone 10 mg per day. She presented predominant abdominal complaints with pain and diarrhea. Physical examination revealed bilateral leg edema and tender abdomen. Biology showed leukopenia with neutropenia (560/mm^3^) and heavy proteinuria (30 g/L). She had active lupus (SLEDAI 10). Her abdominal CT-scan showed edema of the proximal jejunum with target sign and moderate ascites. Treatment consisted of IV methylprednisolone 1 g per day for 3 days, tapered oral prednisone 0.5 mg/kg and long-term HCQ 400 mg per day and MMF 2 g per day with no relapse until present.

#### Case #5

A 28 year-old woman presented in 2008 with a 2-day history of abdominal pain, diarrhea and vomiting. At that time she received low dose corticosteroids (5 mg/day of prednisone), HCQ and Coumadin on a daily basis. She was diagnosed with SLE based on arthritis, serositis, cutaneous involvement, and presence of antinuclear antibodies with positive search for anti-dsDNA antibodies and aPL syndrome at the age of 18 when she presented pulmonary embolism and lupus anticoagulant was detected. Biology was notable for low CH50 and C3 complement levels and INR 4.2. An abdominal CT revealed intestinal edema with target sign and moderate ascites without mesenteric thrombosis. She received 1000 mg IV methylprednisolone for 3 days, with rapid improvement. Because she presented 2 similar episodes over the next 6 months a treatment with MMF and later AZA was installed, however she continued to present chronic intermittent abdominal pain and diarrhea. Multiple repeat imaging and extensive abdominal workup were normal. She is currently treated with HCQ, low dose steroids (5 mg/day of prednisone) and Coumadin and continues to present sporadic abdominal discomfort.

#### Case #6

A 28-year-old woman presented in 2008 with abdominal pain and vomiting. She was diagnosed with SLE 10 years before because of arthritis, lupus nephritis, and presence of antinuclear antibodies with positive search for anti-dsDNA antibodies. She had trouble with compliance and she was not regularly taking medication for the last two years. In retrospect, she mentioned a similar episode 1 year before which had been successfully treated with oral steroids. Biology was remarkable for low complement, lymphocytopenia and significant proteinuria. Abdominal CT revealed small bowel edema with target sign. She was treated with IV methylprednisolone 1 g/day for 3 days with rapid improvement. Oral prednisolone was rapidly tapered and daily HCQ was added. She was subsequently lost for follow up.

#### Case #7

A 48-year-old woman presented in 2010 with a two-day history of abdominal pain and vomiting. She was on daily low dose corticosteroids and HCQ. She had been diagnosed with SLE 2 years before, when she presented with arthritis, cutaneous involvement, cytopenia, and presence of antinuclear antibodies with positive search for anti-dsDNA antibodies. Physical examination was remarkable for abdominal guarding and extensive livedo reticularis. Biology revealed low complement but no argument for hemolysis. Abdominal CT showed edema with target sign of the proximal ileum. She received prednisone 2 mg/kg/day with rapid improvement. Daily MMF was added a few months later, when she presented a severe cutaneous flare because it was felt at that time that she would benefit from additional immunosuppressive therapy as a manner to better control disease activity. She had no recurrence of abdominal pain until present.

### Review

#### Patients’ characteristics

Including the 7 new cases, 150 patients (111 women; 8 men, 31 not mentioned) fulfilling our inclusion criteria for lupus enteritis have been reported in the literature [1,11-48]. Mean age at diagnosis of lupus enteritis was 32.5 years, the youngest patient was 13 and the oldest was 72 years old. Male–female ratio was 1/14. In 19 of the 146 (13%) patients in whom this information was available, SLE was diagnosed simultaneously with the first episode of enteritis. Two patients presented enteritis before diagnosis of SLE [14,20]; one presented a necrotizing vasculitis of the ileum 1 month before diagnosis of SLE, another had lesions in the small intestine on capsule endoscopy 4 months before diagnosis of SLE. When patients were already diagnosed with SLE (n = 126), median time from SLE diagnosis to first episode of enteritis was 60 months (range: 5 months to 20 years).

#### Clinical features

The most frequent symptoms were focal or diffuse abdominal pain (146/150; 97%), ascites (71/91: 78%, clinical or radiological), nausea (36/74; 49%), vomiting (31/74; 42%), diarrhea (24/74; 32%) and fever (15/74; 20%) (Table 2). A surgical abdomen with guarding and rebound was found in 33/105 cases (31%). Four patients had clinically evident digestive bleeding. Fifty-three patients (35%) had other clinical features of active SLE, including lupus nephritis (n = 37), serositis (n = 15), neuropsychiatric manifestations (n = 11), rash (n = 8), arthritis (n = 6), oral ulceration (n = 4) and recent alopecia (n = 1). One patient had concomitant HSV esophagitis that was treated with Acyclovir [46]. The mean SLEDAI score was 14.6 (range: 3–19) for the 64 patients in whom this data was reported. When a long-term treatment for SLE was reported (89 cases), patients were on low dose corticosteroids (median dose of equivalent prednisone: 10 mg per day, range 5–15 mg); five patients were on HCQ while they presented the first episode of enteritis. Two patients (with known lupus) developed enteritis shortly after delivery [26]. No patients presented concomitant pancreatitis and enteritis. None of the patients exhibited signs of concurrent cutaneous vasculitis.

**Table 2:** Clinical, Laboratory & Imaging features of lupus enteritis

**Clinical**	**Laboratory**	**Imaging**
Abdominal pain	ANA, Anti ds-DNA	Bowel abnormalities (edema, target sign, dilated lumen), predominantly jejunum and ileum
Ascites	Low complement	Ascites
Nausea	Moderate elevated CRP	Mesenteric abnormalities (engorgement of mesenteric vessels, comb sign, increased attenuation of mesenteric fat)
Vomiting	Leukopenia, Lymphocytopenia, Anemia, Thrombocytopenia	
Diarrhea	Proteinuria	
Active SLE features		
Surgical abdomen		
Fever		

None of these features are specific. ANA: antinuclear antibodies; Ds-DNA: Double stranded DNA; CRP: C-reactive protein; Target sign: Abnormal bowel wall enhancement; Comb sign: Increased number of visible mesenteric vessels.

#### Laboratory data

When relevant laboratory data were available (n = 48), patients presented hematological anomalies including anemia (52%) (3 had a positive Coombs test), leukocytopenia and/or lymphocytopenia (40%) and thrombocytopenia (21%). Median C-Reactive Protein (CRP) value (reported in 16 cases) was 2.0 mg/dL (range 0–8.2 mg/dL). Hypocomplementemia was reported in 30 of 34 cases (88%). When autoimmune profile was discussed (n = 94), ANA were positive in 92% and ds-DNA in 74%. Anti-RNP was positive in 28%, anti-SSA in 26% and anti-Sm antibodies in 24%. When urinary analysis was mentioned (n = 43), significant proteinuria (>0.5 g/24 h or ++ on dipstick) was present in 47%. Mean serum protein was 5.7 g/dL (17 patients). One patient had renal failure [43]; another had elevated transaminases and amylase [29]. Positive aPL antibodies were detected in 25/84 patients (30%). Seven patients fulfilled diagnostic criteria for the aPL syndrome. One patient had cryoglobulin [31] (type not mentioned); no patient had positive ANCA reported. When analyzed (n = 9), ascites was a sterile exudate, unless perforation had occurred (1 case) [14].

#### Imaging

Abdominal CT scanning was performed in 132 of 150 patients (88%). Other imaging modalities included plain or contrast enhanced abdominal X-ray (22/150 patients, 15%), abdominal echography (10/150, 7%) and mesenteric angiography (5/150, 3%). One patient was diagnosed with lupus enteritis on capsule endoscopy. All patients underwent small bowel imaging using at least one imaging modality. Therefore, in all patients lupus enteritis was diagnosed on the basis of a confirmed diagnosis of SLE, and on both clinical and radiological signs of lupus enteritis, by one or more of the available imaging modalities. The most frequent radiographic anomalies (repeat imaging on recurrence included) were bowel wall edema (161/176, 91% of all episodes; 151/159, 95% of CT images), abnormal bowel-wall enhancement (double halo or target sign) in 68/96 (71%) and dilatation of bowel lumen in 43/176 (24%). Ascites was observed in (71/91, 78%). Mesenteric abnormalities such as engorgement of mesenteric vessels, increased number of visible vessels (comb sign), and increased attenuation of mesenteric fat were present in 68/96 (71%). No patient had evidence for mesenteric thrombosis on CT. One patient presented diminished mesenteric blood flow on angiography, linked by the authors to histologically confirmed small vessel vasculitis [15]. Seventeen percent had digestive endoscopy (25/150), with normal macroscopic findings in 60%. One patient underwent capsule endoscopy, revealing multiple small ulcers and scars [20]. When the distribution of intestinal involvement was detailed, jejunum and ileum were the most frequent implicated segments (83 and 84% respectively), followed by colon (19%), duodenum (17%) and rectum (4%). Four percent (6/150) of patients developed pneumatosis intestinalis. Eight patients presented urinary bladder wall edema (lupus cystitis) concomitant with enteritis, which was linked to an associated hydronephrosis in five.

#### Pathology

Thirty-four patients underwent biopsy, either endoscopic or surgically. Macroscopic findings were described as segmental areas of edematous (n = 8), hyperemic (n = 2) or ischemic (n = 1) bowel with or without ulceration (n = 5), necrosis (n = 3), wall thinning (n = 2) or perforation (n = 1). Two patients had white nodules or plaques on the peritoneal surface [31,47]. Microscopic findings included cellular infiltration of the submucosal (n = 2) and muscular (n = 2) layers with or without edema (n = 3) or vasculitis. Hemorrhage was present in the muscular and subserosal layers in 2 cases. Nine patients [1,11,12,15,16,18,29,37],[48] who underwent biopsy had straightforward histologic vasculitis (26% of biopsies, 9/150, 6% of population), confirmed exclusively on laparotomy. Vasculitis has been characterized as necrotizing vasculitis with fibrinoid necrosis (n = 5), with panmural (n = 1) predominant eosinophilic (n = 2), neutrophilic (n = 1) or mixed (n = 1) infiltrate. Vessels in submucosal (n = 6), muscular (n = 4) and serosal (n = 3) layers as well as mesenteric vessels (n = 2) were affected. Both arterioles and venules (n = 1) could be affected or in contrast a pattern of involvement of veins and venules with sparing of arteries (n = 1) was reported. One patient had vasculitis of the polyarteritis nodosa type (medium size vessels). Microvascular thrombus formation was reported in 3 cases [14,16,47]. One of these patients had aPL antibodies (low positive anticardiolipin); none had diagnostic criteria for the aPL syndrome [49]. Other findings included complement deposition on immunofluorescence in intestinal veins and basement membrane (n = 1) [48].

#### Treatment-outcome

Detailed treatment is shown in Additional file 1: Table S1. All patients received corticosteroids as initial treatment, either intravenous (IV) or oral (PO). IV corticosteroids (122/141, 87 %) in form of methylprednisolone were administered in doses ranging from 40 mg per day over 1 mg/kg per day to 30 mg/kg per day in a fatal pediatric case [19]. Average duration of IV treatment was 4 days, ranging from 1 to 34 days, commonly until clinical improvement was obtained. When oral steroids were chosen as initial treatment (19/141 13%), prednisolone was delivered in doses ranging from 20 mg per day to 2 mg/kg per day, tapered over several weeks to months to a median daily maintenance dose of 5 mg (range 5–20 mg). Ninety-four (141/150) percent of patients received corticosteroids alone. Additional immunosuppression on initial treatment (n = 11) consisted of PO (n = 1) or IV cyclophosphamide (CYC) at 500 mg/m^2^ to 750mg/m^2^. This was justified by concomitant severe organ involvement in 5 (central nervous system involvement in 2 [32,43], lupus nephritis in 3 [18,47]), associated intestinal necrosis in 4 [12,19,29,35,37] and persistent abdominal pain despite of IV steroids in one patient [12]. Seventeen patients underwent laparotomy, of which 10 (7%) had resection for necrosis or perforation.

#### Evolution

Relief of symptoms took typically less than a week (range: 2 days to 8 weeks), with parallel biological and radiological normalization. Long-term maintenance therapy after a first episode (other than prednisolone) consisted of HCQ (n = 7), MMF (n = 3), azathioprine (AZA) (n = 2) and oral chlorambucil (n = 1).

Recurrence, i.e. more than one episode of enteritis, was reported in 34 patients (23%) with a median number of 3 episodes (range: 2–9). When we considered patients with reported observance of at least 1 year (n = 30), recurrences occurred in 25%. On recurrent episodes, corticosteroids were used similar to first episodes. Additional immunosuppression consisted of IV CYC (n = 4) [1,25,43,44], MMF (n = 4) [25,34,44], AZA (n = 4) [25,34,43], Rituximab (n = 2) [34,44] and PO CYC (n = 1) [34]. Declared morbidity included short bowel syndrome with home total parenteral nutrition (n = 1) [37], recurrent abdominal bloating and dyspepsia (n = 1) [25] and recurrent abdominal pain without imaging abnormalities (n = 2). Four patients died after a median follow-up of 18 months, yielding a mortality rate of 2.7%. Reported causes of death included diffuse necrosis of intestinal tract (n = 1) [14], associated neurologic complications (n = 1) [11] and sepsis (n = 1) [19].

## Discussion

Lupus enteritis is a potentially severe complication of SLE, stressing the need for swift diagnosis and adequate management. The literature on the subject consists entirely out of case reports and case series, and no controlled studies on treatment are yet available. Here, we have reported 7 new cases of lupus enteritis and thoroughly reviewed 143 cases from the literature to draw out recommendations for the diagnosis and management of this unusual complication of SLE.

The clinical picture of lupus enteritis is non-specific, with abdominal pain being the cardinal manifestation, sometimes accompanied by symptoms and signs of impaired intestinal motility or peritonitis. Other elements of active lupus [50] are universally present. Buck et al. [51] found that only patients with active disease (SLEDAI score >8) and acute abdominal pain were diagnosed with “lupus mesenteric vasculitis”, but Lee et al. [30] found no difference in SLEDAI scores between lupus enteritis and other causes of abdominal pain in 175 lupus patients, suggesting that SLEDAI score is not suited for differencing the cause of abdominal pain in active lupus. It is noteworthy that the SLEDAI score contains no items to score abdominal SLE disease activity, in contrast to the British Isles Lupus Assessment Group (BILAG) index [52].

CT scanning has become the gold standard for diagnosis of lupus enteritis. One should keep in mind that in SLE patients, who are commonly treated with corticosteroids, signs of perforation might be clouded; consequently, the threshold for radiographic evaluation should be low. Typical features of lupus enteritis include focal or diffuse bowel-wall thickening, bowel dilation, abnormal bowel wall enhancement (target sign), engorgement of mesenteric vessels with increased number of visible vessels (comb’s sign), increased attenuation of mesenteric fat and ascites [15,27]. Bowel wall involvement is mostly multisegmental and not confined to a single vascular territory [1], with jejunum and ileum being the most commonly involved sites. However, the lack of specificity of CT signs is a limitation, as the above described abnormalities can also be seen in patients with pancreatitis, mechanical bowel obstruction, peritonitis, or inflammatory bowel disease, all of which may mimic intestinal ischemia [53]. Abdominal ultrasound seems to be an elegant tool to confirm bowel edema or ascites and might be useful to confirm straightforward clinical diagnosis or alternatively in follow-up to affirm clinical recovery. Endoscopic biopsies are not very rewarding, possibly because only superficial tissue is obtained or due to sample effect. We suggest that these should be used primarily to confirm or rule out alternative diagnoses rather than to confirm vasculitis. None of our 7 new cases underwent biopsy of the affected intestinal tract.

No single biological finding can be considered pathognomonic for lupus enteritis. CRP is typically not very elevated and high CRP should be considered suggestive for an alternative cause of abdominal pain or infectious complications [54]. Twenty-eight percent of patients with lupus enteritis had positive aPL biology, which is compatible with the background prevalence of aPL antibodies in SLE [55]. Three patients had thrombosis on histology and only one of them had weak positive anticardiolipin antibodies. Two studies [1,30] found no differences in autoantibody profiles including those of aPL antibody between SLE patients with lupus enteritis and other causes of abdominal pain. Thus it seems unlikely that thrombosis in the context of the aPL syndrome is involved in lupus enteritis.

In the BILAG 2004, lupus enteritis is defined as either vasculitis or inflammation of the small-bowel. Although lupus enteritis is commonly considered a form of visceral or serosal vasculitis, this is seldom confirmed on histology. Only 9 patients (6%) had clear histological confirmation of vasculitis. Drenkard [56] reported only one case of mesenteric vasculitis among 667 patients with vasculitis in SLE. Consequently, we advocate the use of the generic term enteritis rather than vasculitis. Other than being merely a non-specific biological sign of active SLE, the frequently encountered hypocomplementemia in lupus enteritis might be a consequence of immune-complex-mediated vasculitis in the development of lupus enteritis [57]. In the single case where immunofluorescence was reported, complement deposition in mesenteric small vessels was found [48]. Independent of potential pathogenesis, events eventually lead to edema, bleeding and necrosis, translating into clinical symptomatology of pain, diarrhea, bleeding, obstruction and perforation. Peritoneal irritation, whether primary (serositis) or secondary (intestinal vasculitis), can lead to ascites [58].

Lupus enteritis is generally reversible and steroid-responsive. Previous studies [1] failed to identify factors that could predict therapy-resistant or relapsing forms of enteritis. Early surgical intervention rather than steroid therapy as a treatment for lupus enteritis has been advocated [6], but does not seem legitimate in view of high steroid responsiveness, reversibility and limited morbidity and mortality. We would rather encourage extreme vigilance for perforation and peritonitis with low threshold for (repeat) CT imaging and early laparoscopy or laparotomy to evaluate intestinal viability in case of doubt. Abdominal paracentesis for ascites does not seem to be very useful unless there is doubt about its etiology or as a therapeutic measure if abundant.

**Figure 2:**
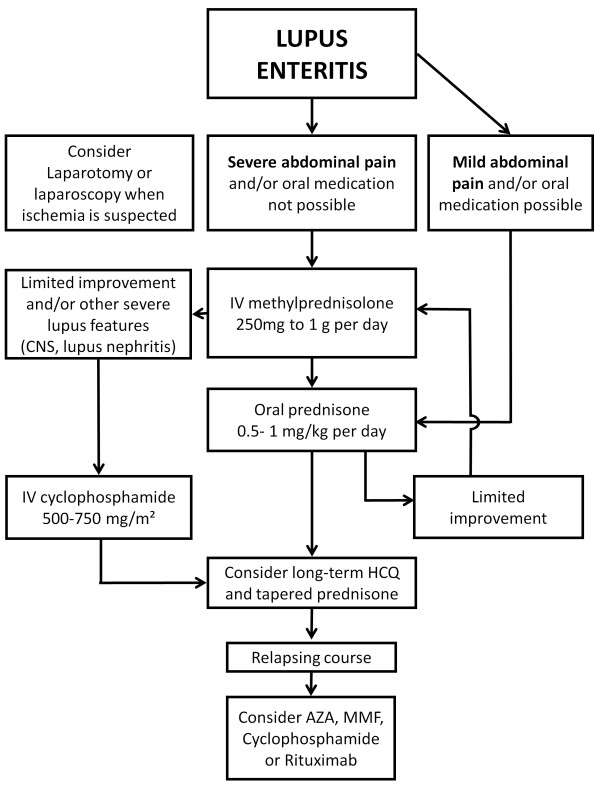
**Therapeutic strategy for lupus nephritis.** CNS: central nervous system; IV: intravenous; HCQ: hydroxychloroquine; AZA: azathioprine; MMF: mycophenolate mofetil.

Well-aware of the absence of any prospective controlled studies on treatment of lupus enteritis but merely synthesizing therapies applied in the considered papers we propose a therapeutic strategy, ideally to be tested in future controlled studies (Figure 2). Based on the high steroid-responsiveness, corticosteroids appear to be the first line treatment of lupus enteritis. These may be administered IV or PO based on clinical status or other organ involvement, with preference for IV in case of severe lupus flare because of potentially reduced drug absorption due to enteritis [59]. CYC or MMF might be added in case of resistance to corticosteroids or when warranted by other organ involvement. Treatment can be switched to oral corticosteroids as soon as adequate clinical improvement occurs, with tapering according to clinical evolution. HCQ, MMF, AZA and low dose corticosteroids could be considered for long-term maintenance treatment, although it is unknown whether they may prevent recurrences. In recurrent forms MMF, AZA, CYC and Rituximab have been successfully used to prevent further recurrence, in a limited number of cases. In absence of controlled studies [60] the choice for a particular immunosuppressive regimen should be based on individual benefit-risk ratio, considering other organ involvement or a potential pregnancy wish. Supportive measures include bowel rest, IV fluids and proton pump inhibitors. Heparin might be added in presence of aPL antibodies and suspicion of aPL syndrome. Early laparoscopy or laparotomy should be considered if necrosis or perforation is suspected.

## Conclusion

Lupus enteritis is a rare complication of SLE. Diagnosis is based on typical CT findings (bowel wall edema with target sign, mesenteric abnormalities and ascites) in SLE patients with acute abdominal pain. Lupus enteritis is typically steroid-responsive with an overall excellent prognosis. Immunosuppressive treatment is reserved for recurrent enteritis or severe SLE cases. We advocate simplifying nomenclature by the use of the single term “lupus enteritis” and we propose better-defined diagnostic criteria to limit heterogeneity. Finally, we stress the need for a prospective evaluation of this rare disease by encouraging establishment of an international register.

## Abbreviations

ACR: American College of Rheumatology; ANA: Antinuclear antibodies; ANCA: Anti-neutrophil cytoplasmic antibodies; aPL: Antiphospholipid; BILAG: British Isles Lupus Assessment Group; CRP: C-reactive protein; CYC: Cyclophosphamide; ds-DNA: Double stranded DNA; HCQ: Hydroxychloroquine; HSV: Herpes simplex virus; IV: Intravenous; MMF: Mycophenolate mofetil; PO: Oral; SLE: Systemic Lupus Erythematosus; SLEDAI: Systemic Lupus Erythematosus Disease Activity Index; TSH: Thyroid stimulating hormone.

## Competing interests

The authors’ declared that they have no competing interests.

## Authors’ contributions

PJ, LA, LG, AM, MH, DS, JH, CVB, JCP and ZA collected clinical data. IHC and PAG performed imaging studies. PJ, LA, LG and ZA wrote the manuscript. All authors read and approved the final manuscript.

## Supplementary Material

Additional file 1: Table S1 Detailed treatment. Click here for file
