# Supplementary material for: Lupus enteritis: from clinical findings to therapeutic management
Source: Orphanet J Rare Dis. 2013 May 3;8:67. doi: 10.1186/1750-1172-8-67 (PMC3651279; doi:10.1186/1750-1172-8-67)
Supplement: Additional file 1: Table S1 — Detailed treatment. [file 1750-1172-8-67-S1.doc]

**Table S1: Detailed treatment**

| **Author (year) ref number** | **Initial Treatment** | **Maintenance** | **Recurrence** | **Outcome** |
| --- | --- | --- | --- | --- |
| **Thomas (2010)^9^** | Prednisone PO 60 mg/d | HCQ, prednisone 15 mg/d |  | Quick resolution of symptoms. No recurrence after 1 year. |
|  | Prednisolone PO 20 mg/d | HCQ, low dose prednisone |  | Resolution of symptoms with no recurrence after 1 year. |
|  | Methylprednisolone IV 200 mg/d for 3 days, tapered oral prednisolone |  |  | Lost for follow up |
| **Oh (2010)^10^** | Prednisolone PO 1 mg/kg/d | MMF | 8 times. Methylprednisolone alone IV 1mg/kg/d on 3 occasions, MMF (relapse after 1month), AZA (relapse after 4m), oral CYC (relapse after 3m), Rituximab 500 mg 3 times over 15m (1 recurrence 3 w after first dose); Oral prednisolone tapered in 15 m | No recurrence after 3 cycles Rituximab |
| **Marinello (2O1O)^11^** | Methylprednisolone IV 1g/d, IV CYC because of central nervous system involvement | Oral prednisolone and monthly CYC |  | Normalization CT 10 days after initiating treatment |
| **Wakui (2010)^12^** | Methylprednisolone IV 500 mg/d for 3 days. Prednisolone 40 mg/d for 1m, tapered to 20mg/d in 3m. |  |  | Symptoms gradually improved |
| **Huang (2009)^13^** | Methylprednisolone 2mg/kg/d, tapered over 2 weeks | Low dose prednisolone and AZA |  |  |
| **Tu (2009)^14^** | Methylprednisolone IV 1g/d for 3 days, CYC 500 mg/m^2^ for concomitant seizures | Prednisolone 2,5 mg for 2 years |  | Abdominal symptoms improved after 3 days |
|  | Hydrocortisone IV 10 mg/kg/d | Oral prednisolone | Several recurrences, finally controlled after 5 courses IV CYC (0,5-1g/m^2^) Long term prednisolone 15 mg/d and AZA 50 mg/d |  |
|  | Hydrocortisone IV 10 mg/kg/day, prednisolone 1mg/kg PO when abdominal pain subsided | Long term prednisolone PO10 mg/d |  |  |
| **Saito (2008)^15^** | High dose steroids, anticoagulants, IV CYC, resection of the ileum |  |  | Short bowel syndrome |
| **Assimakopoulos (2008)^16^** | Methylprednisolone IV and CYC IV (highly active lupus) |  |  | Symptoms resolved over 5 days |
| **Mizoguchi (2008)^17^** | Prednisolone PO 60 mg/d for 1m, IV CYC 750 mg monthly (reason?) |  |  | Immediate improvement, development of pneumatosis intestinalis 1m later (treated with hyperbaric oxygen and prokinetics), resolution after 2m |
| **Kwok (2007)^1^** | Forty-three patients. Methylprednisolone IV 1-2mg/kg/d for 3 days, PO prednisolone thereafter |  | Twelve patients had recurrence, all treated with IV and PO corticosteroids; 1 patient had monthly IV CYC to prevent further recurrence | One patient required intestinal resection due to infarction |
| **Waite (2007)^18^** | Each time IV and PO steroids | MMF | MMF ineffective; two cycles of methylprednisolone IV 1OO mg, rituximab IV 5OOmg, CYC IV 500mg | Each time good response on IV steroids; gram negative sepsis between the 2 cycles of additional immunosuppression; two years without relapse thereafter |
| **Kishimoto (2007)^19^** | Methylprednisolone IV 40 mg 3/d during acute crises |  | Nine times; always methylprednisolone IV during acute crises. Sixteen cycles IV CYC, AZA PO 150 mg/d with recurrence; MMF PO 1000 mg/d, recurrence 1 more time | Occasional abdominal bloating, no recurrence of pain |
|  | Laparoscopy ruled out necrosis, methylprednisolone IV 40 mg 4/d for 3 days, prednisolone PO 25mg 2/d |  | Two recurrences; high dose  Methylprednisolone IV followed by tapered prednisone PO | Rapid improvement of symptoms with steroids |
| **Endo (2007)^20^** | Prednisolone PO 30 mg/d |  |  | The symptoms improved promptly with steroids. Developed of a malar rash on prednisolone reduction |
| **Sunkureddi (2005)^21^** | Prednisone PO 40 mg/d, tapered to 5 mg/d in 8 weeks | HCQ |  | Eight weeks later the patient had no symptoms |
| **Kaneko (2004)^22^** | Prednisolone PO 17,5 mg/d and CYC PO 50mg/d |  | Seven times; treated by increasing prednisone to 15 à 45 mg/d | Each time improvement of enteritis within a few days |
| **Passam (2004)^23^** | Laparotomy with resection, 2 days later extended resection of the ileum; prednisolone IV 1mg/kg/d and IV heparin | 12 cycles CYC IV 20 mg/kg; prednisolone PO 20 mg/2d |  | 2 years later in remission |
| **Chung (2003)^24^** | Prednisolone PO 20mg/d | AZA PO 50 mg/d |  | Prompt resolution of symptoms on steroids |
| **Lee (2002)^25^** | Seventeen patients. Methylprednisolone IV 1mg/kg/d followed by tapered oral prednisolone |  | Four patients relapsed and were treated with IV steroids | All responded well to IV treatment |
| **Alcocer (2000)^26^** | High dose steroids IV, laparotomy after 10 days, CYC 500mg/m2 postoperative |  |  | Discharged 38 days postoperative |
| **Weinstein (2000)^27^** | Exploratory laparotomy with resection of the appendix; methylprednisolone postoperatively; CYC was added when proteinuria and renal insufficiency developed |  |  | The patient improved, and was discharged with minimal ascites and mild renal insufficiency |
| **Byun (1999)^28^** | Thirty-one patients. Methylprednisolone IV, average dose 164 mg/d, from onset to improvement of symptoms. Oral tapered steroids; one patient had laparotomy without resection for clinical peritonitis |  | Six patients had recurrence; | One patient had segmental resection of the jejunum because of bowel infarction 6m after remission |
| **Hizawa (1998)^29^** | Four patients. Prednisolone PO 1mg/kg/d |  |  | Symptoms relieved in less then 7 days |
| **Ko (1997)^30^** | Eleven patients. High dose hydrocortisone IV (500mg 2-4/d) for an average of 8.4 days, until satisfactory clinical improvement; two patients had initial high dose prednisone PO and were then switched to IV |  |  | All had normalization of imaging within 12 days |
| **Tsushima (1996)^31^** | Prednisolone 40 mg IV |  |  | Normalization of CT after 1 week |
| **Wakiyama (1996)^32^** | Exploratory laparotomy, postoperatively methylprednisolone IV 1g/d for 3 days switched to prednisolone PO 80 mg/d, tapered to 40 mg/d in 2 weeks |  |  | Uneventful recovery |
| **Low (1995)^33^** | Laparotomy, high dose steroids |  |  | Initial remission but long term outcome uncertain |
| **Cabrera (1994)^34^** | Resection of the distal jejunum and ileum; prednisone PO 80mg/d postoperative | Prednisone PO 20 mg/d and HCQ |  |  |
| **Kirshy (1991)^35^** | Laparotomy without resection; high dose steroids IV |  |  | Complete normalization on imaging 2 weeks later |
| **Eberhard (1991)^36^** | Methylprednisolone 1.5mg/kg/d, laparotomy on day 41 for severe abdominal pain with resection of perforated jejunum; postoperative methylprednisolone IV 30mg/kg/d for 3 days and CYC IV 1mg/kg/d; prednisolone PO 80-120mg for 6 weeks |  |  | The postoperative course was protracted and complicated. The patient died the 95th day of septic shock |
| **Decrop (1990)^37^** | Steroid dose was raised, CYC was added because of renal deterioration; day 28 resection of small bowel segments |  |  | The patient died 18th day postoperatively of cardiorespiratory failure |
| **Laing (1988)^38^** | Methylprednisolone KV 1g/d for 3 days, switch to prednisolone PO 60mg/d, laparotomy with resection of the proximal jejunum on day 14; first cycle CYC IV postoperatively | Nine cycles CYC IV, after 1Y oral chlorambucol in an effort to eliminate the need for monthly CYC |  |  |
| **Knecht (1985)^39^** | Prednisone PO 60 mg/d |  |  | Improvement over a few days |
| **Bringer (1981)^40^** | Laparotomy with ileum resection; further resection a few weeks later. IV methylprednisolone followed by prednisolone PO 2 mg/kg/d | HCQ |  | Ten weeks later catastrophic intestinal necrosis, the patient died postoperatively |
| **Weiser (1981)^41^** | High dose steroids |  |  | Slow improvement over 8 weeks |
| **NEJM 25-1978 (1978)^42^** | Laparotomy with resection ileum; postoperative steroids |  |  | Initial amelioration but development of pericarditis, died 30^th^ day postoperative of neurologic complications |
| **Stoddard (1978)^43^** | Laparotomy without resection; hydrocortisone IM 400 mg 2 weeks, then prednisolone PO |  |  | Recovery was slow with further attacks and delayed return of alimentary function |
| **Shapeero (1974)^44^** | Steroids |  |  | Within days cessation of pain |
| **Kurlander (1964)^45^** | Prednisone PO 60 mg/d gradually decreased |  |  | Improvement on steroids |
| **Pollak (1958)^46^** | Exploratory laparotomy without resection, ACTH and later cortisone |  | Five more times, treated with cortisone PO 75 to 150 mg/day on each occasion | Symptoms subsided over a period of days to weeks |

HCQ: Hydroxychloroquine. MMF: Mofetil Mycofenolate. AZA: Azathioprine. CYC: Cyclophosphamide
